# Genomic partitioning of growth traits using a high-density single nucleotide polymorphism array in Hanwoo (Korean cattle)

**DOI:** 10.5713/ajas.19.0699

**Published:** 2020-01-13

**Authors:** Mi Na Park, Dongwon Seo, Ki-Yong Chung, Soo-Hyun Lee, Yoon-Ji Chung, Hyo-Jun Lee, Jun-Heon Lee, Byoungho Park, Tae-Jeong Choi, Seung-Hwan Lee

**Affiliations:** 1 Division of Animal and Dairy Science, Chungnam National University, Daejeon 34134, Korea; 2 Animal Genetic Improvement Division, National Institute of Animal Science, RDA, Seonghwan 31000, Korea; 3 Department of Beef Science, Korean National College of Agriculture and Fisheries, Jeonju 54874, Korea; 4 Poultry Science Division, National Institute of Animal Science, RDA, PyeongChang 25342, Korea

**Keywords:** Genetic Architecture, Genome-wide Association Study, Hanwoo

## Abstract

**Objective:**

The objective of this study was to characterize the number of loci affecting growth traits and the distribution of single nucleotide polymorphism (SNP) effects on growth traits, and to understand the genetic architecture for growth traits in Hanwoo (Korean cattle) using genome-wide association study (GWAS), genomic partitioning, and hierarchical Bayesian mixture models.

**Methods:**

GWAS: A single-marker regression-based mixed model was used to test the association between SNPs and causal variants. A genotype relationship matrix was fitted as a random effect in this linear mixed model to correct the genetic structure of a sire family. Genomic restricted maximum likelihood and BayesR: A priori information included setting the fixed additive genetic variance to a pre-specified value; the first mixture component was set to zero, the second to $0.0001\times \sigma_{g}^{2}$, the third $0.001\times \sigma_{g}^{2}$, and the fourth to $0.01\times \sigma_{g}^{2}$. BayesR fixed a priori information was not more than 1% of the genetic variance for each of the SNPs affecting the mixed distribution.

**Results:**

The GWAS revealed common genomic regions of 2 Mb on bovine chromosome 14 (BTA14) and 3 had a moderate effect that may contain causal variants for body weight at 6, 12, 18, and 24 months. This genomic region explained approximately 10% of the variance against total additive genetic variance and body weight heritability at 12, 18, and 24 months. BayesR identified the exact genomic region containing causal SNPs on BTA14, 3, and 22. However, the genetic variance explained by each chromosome or SNP was estimated to be very small compared to the total additive genetic variance. Causal SNPs for growth trait on BTA14 explained only 0.04% to 0.5% of the genetic variance

**Conclusion:**

Segregating mutations have a moderate effect on BTA14, 3, and 19; many other loci with small effects on growth traits at different ages were also identified.

## INTRODUCTION

High-density single nucleotide polymorphism (SNP) panels are available as a result of genome sequencing, and large-scale genome-wide association studies (GWASs) have been conducted to identify many novel loci associated with complex polygenic traits in humans and livestock. A good example is a GWAS for human height, which is a polygenic trait. The heritability of human height is approximately 80% [1,2]. However, conventional genome-wide single-marker association studies explained only a very small portion of heritability for human height [3,4]. Recent GWASs for human height identified 697 variants in 423 loci from large-scale data (>253,000 individuals), but those loci only explained 16% of the entire heritability. Yang et al [5] estimated heritability from 3,925 unrelated individuals and 294,831 SNPs using a linear mixed model to overcome the problem of the “missing heritability.” That study showed that ~45% of variance could be explained by all of SNPs simultaneously. Therefore, most of the presumably missing heritability was not actually missing, but it was not detected because the single SNP effects were too small to pass stringent statistical tests [5]. Therefore, in order to search for markers that can increase the explanatory power of genetic variance, it is necessary to obtain a large number of markers and confirm their association.

Nowadays, as the sequencing information of livestock was released and SNP array was developed, GWASs can consider that addresses three objectives of the livestock industry. First, GWASs aim to identify genomic regions, such as quantitative trait loci (QTL), harboring causal variants underlying genetic variation in polygenic traits. Second, GWASs are used to investigate the genetic architecture of polygenic traits by estimating the genetic variance explained by a genomic region containing causal variants. Finally, GWASs can be used to predict genomic estimated breeding value and future phenotypes to rank animals for selective breeding [6]. Growth traits are associated with profit in livestock raised for meat production. Therefore, growth traits are an important breeding trait for selection in Hanwoo Korean cattle breeding programs [7]. Growth traits are genetically highly correlated with carcass weight (CWT), which has a large contribution to total auction price in the Hanwoo production system.

The previous genome-wide QTL mapping studies have identified a major QTL for bovine body stature and growth traits on chromosome 14 [8,9]. Karim et al [8] localized a major genomic region for bovine body stature to a 780-kb segment on bovine chromosome 14, which contains two candidate quantitative trait nucleotides (QTNs) located at PLAG1–CHCHD7. Nishimura et al [9] identified three QTL regions for CWT on bovine chromosomes 6, 8, and 14 in Japanese Black cattle, and the QTL mapped on BTA14 was the same region as a PLAG1–CHCHD7 QTN for stature. Based on these previous studies, it can be estimated that the genomic relationship of growth and CWT traits can be confirmed in Hanwoo cattle.

Recent developments in bovine high-density SNP arrays, such as the BovineSNP50 BeadChip (Illumina, San Diego, CA, USA), allow for an understanding of the genetic architecture of complex traits and to more accurate prediction of genomic breeding value in livestock. Moreover, genomic prediction using genome-wide SNP markers is a promising breeding technology to accurately predict breeding value at an early stage of identifying candidate animals for selection [10]. The genetic architecture of polygenic traits, such as the number of SNPs associated with the polygenic traits and the distribution of SNP effects can affect the accuracy of genomic prediction [11,12]. In this point of view, GWASs can help to clarify the genomic structure of polygenic traits for the livestock industries. Hayes et al [12] performed a GWAS to determine the number of SNPs associated with coat color and milk fat percentage and the distribution of SNP effects for more accurate prediction of breeding value. Therefore, it is necessary to estimate more accurate breeding value in order to efficiently improve Hanwoo, and the causal mutation or genetic marker searched by GWAS analysis of production related trait and genomic information can provide useful information for estimating more accurate breeding value.

The aim of the present study was to characterize the number of loci affecting growth traits and the distribution of SNP effects on growth traits using a whole-genome association study in Hanwoo cattle.

## MATERIALS AND METHODS

### Animals and genotype assays

The animals (n = 904) used in the study were born from 2000 to 2010 and belonged to the National Progeny test program (only male samples). Phenotype data were live body weight (BW) at different ages (6, 12, 18, and 24 months). The number of genotyped individuals and phenotypic growth traits and the trait summary statistics and heritabilities are shown in Table 1. The average BW±standard deviation was 164.3±28.2 kg at 6 months (BW6), 310.7±33.9 kg at 12 months (BW12), 475.4±45.3 kg at 18 months (BW18), and 618.0± 58.6 kg at 24 months (BW24). The heritability for growth traits was 0.25, 0.32, 0.38, and 0.45 for BW6, BW12, BW18, and BW24, respectively (Table 1). DNA was extracted from blood using the DNeasy 96 Blood & Tissue Kit (Qiagen, Valencia, CA, USA). DNA quality control (QC) was assessed and DNA quantified using the NanoDrop 2000 (Thermo Fisher Scientific, Inc., Wilmington, DE, USA). Total DNA concentration was diluted to 900 ng with a 260/280 ratio >1.9, and the final DNA concentration for genotyping was 20 ng/μL. The high-density SNP genotyping was performed using the Illumina bovine 50K array (Illumina, USA). All genotyping was done by the Animal Genome & Bioinformatics Division of the National Institute of Animal Science, RDA, Korea. As a result of the genotyping of the 50k array, a total of 48,845 SNPs was obtained, which was imputed to the 777K bovine reference genome (731,261) SNPs using 4,887 reference population. The SNP QC criteria were call rate of 90%, minor allele frequency (MAF) <0.01, and Hardy–Weinberg equilibrium (HWE) <0.0001. SNP QC was performed using Plink1.9 software.

### Genome-wide association to reveal the genetic architecture of complex traits in Hanwoo cattle

A single-marker regression-based mixed model was used to test the association between the SNPs and the causal variants. A genotype relationship matrix (GRM) was fitted as a random effect in this linear mixed model to correct the genetic structure of the sire family. The basic concept of a GWAS is that the SNP is assumed to be in linkage disequilibrium (LD) with causal variants in close proximity. The effect evaluated was an additive effect. SNP genotypes were recoded as allele counts (0, 1, 2), which represent copies of the variant allele. The following mixed linear regression model was fitted to map QTL for growth traits using a genome-wide complex trait analysis (GCTA) [5]. The statistical model was,

$$Y_{\mathrm{ijkl}}=\mu +{\mathrm{CG}}_{i}+b_{1}{\mathrm{Sage}}_{j}+b_{2}{\mathrm{SNP}}_{k}+A_{l}+e_{\mathrm{ijkl}}$$

where ***Y****_ijkl_* is the phenotype of the growth traits, *μ* is the overall mean, ***CG****_i_* is a contemporary group for birth year and season and test-round effects, ***b*_1_** is a regression coefficient, ***Sage****_j_* is slaughter age as a covariate, ***b*_2_** is genotypic effect, ***SNP****_k_* is SNP genotype (0, 1, 2), ***A****_l_* is polygenic as a random effect $\sim \text{N }(0,G\sigma_{a}^{2})$, and **e** is a residual effect as a random $\sim \text{N }(0,I\sigma_{a}^{2})$.

### Genomic partitioning of the growth traits in using genomic restricted maximum likelihood in Hanwoo cattle

Two genomic regions were identified on BTA14 and 3, that may harbor causal variants of large genetic effect. We estimated the GRM of all individuals from autosomal SNPs using GCTA software to partition the genetic variance explained by restricted maximum likelihood analysis in each chromosome [5]. The statistical model was,

y=Xβ+gG+e,

where ***y*** is a vector of the growth traits, ***β*** is a vector of fixed effects of the contemporary group (birth year, season, and test round effects), ***X*** is an incidence matrix, ***gG*** is a vector of genetic effects of all autosomal SNPs with $\mathrm{var}(\mathrm{gG})=A_{g}\sigma_{G}^{2}$, and ***A****_g_* is the GRM for all autosomal SNPs. The proportion of variance explained by all autosomal SNPs was defined as $h_{G}^{2}=\sigma_{G}^{2}/\sigma_{P}^{2}$. Furthermore, we estimated the GRM from the SNPs on each chromosome and estimated the variance attributable to each chromosome by fitting the GRMs of all chromosomes simultaneously in the linear mixed model

$$y=X\beta +\Sigma_{\mathrm{chr}=1}^{29}\;\mathrm{gc}+e,$$

where ***gc*** is a vector of genetic effects to each chromosome, and $\mathrm{var}(\mathrm{gc})=A_{c}\sigma_{c}^{2}$ (joint analysis). The proportion of variance explained by each chromosome was defined as $h_{G}^{2}=\sigma_{C}^{2}/\sigma_{P}^{2}$. We then plotted the heritability of each chromosome to investigate genomic partitioning for growth and the average daily weight gain trait in Hanwoo cattle.

### Bayesian mixture model analysis

*A priori* conditions included setting the fixed additive genetic variance to a pre-specified value, such as setting the first mixed component to zero, the second to $0.0001\times \sigma_{g}^{2}$, the third $0.001\times \sigma_{g}^{2}$, and the fourth to $0.01\times \sigma_{g}^{2}$. Bayesian mixture model (BayesR) fixed a priori information at not more than 1% of the genetic variance for each SNP effect using a mixed distribution. The BayesR model set the a priori condition that if the number of markers was not in LD with the causal variant, then these have zero effect, whereas markers associated with a causal mutation have a small to moderate effect. Therefore, the SNP effects are conditionally related to the variance of the components $\sigma^{2}=(\sigma_{1}^{2},\cdots ,\sigma_{k}^{2})$ and the mixed proportions ***π*** = (***π*_1_**, ⋯, ***π****_k_*) which are constrained to be positive and to sum to unity: $p(\beta |\pi ,\sigma^{2})=\Sigma_{k=1}^{k}\pi_{k}N(\beta |0,\sigma_{k}^{2})$, where ($\beta |0,\sigma_{k}^{2}$) indicates the density function of the univariate normal distribution with mean 0 and variance $\sigma_{k}^{2}$. The Bayesian approach requires the assignment of prior distributions to all unknowns in the model. Following Erbe et al [13], we assumed an a priori mixture of four zero-mean normal distributions, where the relative variance for each mixture component was fixed:

$$p(\beta |\pi ,\sigma^{2})=\pi_{1}\times N(0,0\times \sigma_{g}^{2})+\pi_{2}\times N(0,10^{-4}\times \sigma_{g}^{2})+\pi_{3}\times N(0,10^{-3}\times \sigma_{g}^{2})+\pi_{4}\times N(0,10^{-2}\times \sigma_{g}^{2})$$

Here, ***σ*^2^** is the additive genetic variance explained by SNPs that fixed the marker variance at a pre-specified value.

## RESULTS AND DISCUSSION

**Figure 1:**
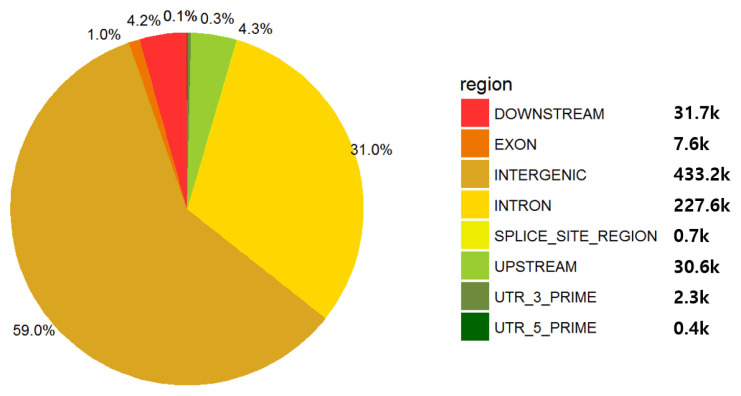
The SNP annotation result of imputed 777K SNPs to bovine reference genome. Of the total 644,726 SNPs completed, approximately 90% of the 660.8K variants were identified as intergenic and intron SNPs, and about 1% of the 7.6K SNPs were identified as exonic SNPs. SNP, single nucleotide polymorphism.

The 50K SNP information obtained for GWAS and genomic partitioning analysis using growth traits and genomic information was imputed as follows. A total of 731,261 SNPs was imputed using 48,845 samples of 777K SNPs as a reference. After QC assessment, 644,726 SNPs were included for further analyses, and 782, 82,450, and 4,085 SNPs were removed by QC criteria of low call rate, MAF, and HWE, respectively. The majority of SNPs were mapped to the intergenic and intron regions, i.e., 59% and 31% of SNPs were located in intergenic and intron regions of the bovine reference genome, respectively (Figure 1). The exon region mapped only 1% of SNPs, and the regulatory and upstream regions included 4.3% (Figure 1).

### Genome-wide association results for each growth traits

**Figure 2:**
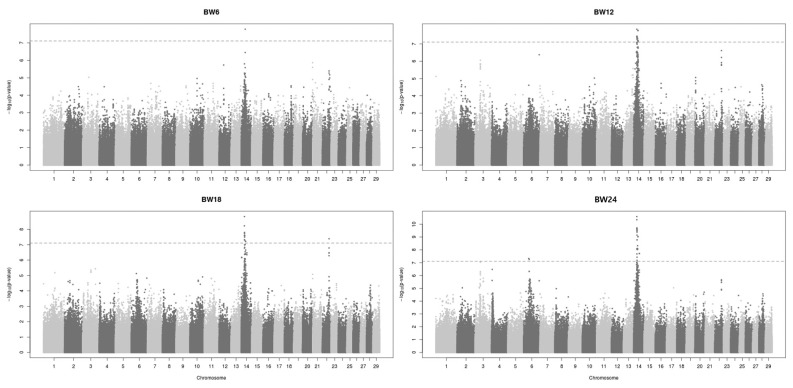
Genome wide association study for growth traits using linear mixed model in Hanwoo. A total of 72 regions in all BW measurement intervals were identified as positions representing the association with each growth trait. Among them, 38 SNPs showed association with growth traits except for overlapping SNPs. BW6 has 1 SNP on BTA14, BW12 has 17 SNPs on BTA14, BW18 has 19 SNPs on BTA14 and 1 SNP on BTA22, BW24 has 32 SNPs on BTA14 and 2 SNPs on BTA6 were confirmed association results with each growth trait. BW, body weight; SNP, single nucleotide polymorphism.

The GWAS revealed that common 2-Mb genomic regions may contain a causal variant on BTA14 for body weight at 6, 12, 18, and 24 months (Figure 2). This genomic region explained approximately 10% of the genetic variance against the total additive genetic variation. A total of 72 SNPs showed significant association with BW traits at each stage, and 69 of them were found on BTA14. In this chromosome, the 22 SNP regions were identified on 24 to 27 Mbp range. These areas were identified as matching areas in the phenotypes at BW12, 18, and 24 months. (Supplementary Table S1). Other than this region, the genomic region on BTA22 (48M) showed a significant effect on body weight at 18 months. Two SNP positions (39M and 42M) on BTA6 showed significant effects for body weight at 24 months in Hanwoo. The body weight trait is genetically correlated with CWT in cattle. A recent GWAS of Hanwoo cattle identified a highly significant genomic region for CWT on BTA14 [14,15]. That study detected a major genome region ranging from 23 to 25 Mb on BTA14 associated with CWT in Hanwoo. The most significant SNPs within the region explained 6.7% to 10.6% of the genetic variance, which is a large proportion of the total additive genetic variance. In cattle other than Hanwoo, BTA14 is well known as a major QTL region for bovine body stature, CWT, and growth traits [8,9]. Karim et al [8] revealed that the major QTL for bovine stature was localized to a 780 kb section on BTA14 and was mapped to two candidate genes (PLAG1–CHCHD7). Moreover, Nishimura et al [9] reported three major genomic regions for CWT and body stature on BTA6, 8, and 14 in Japanese Black cattle. Taken together, previous studies suggest that BTA14 has a high genetic relationship to growth and CW traits and a high probability of finding causal variation. Thus, a high-density bovine SNP array allowed animal scientists to understand the genetic architecture of a polygenic trait.

### Genomic partitioning of the growth traits

**Figure 3:**
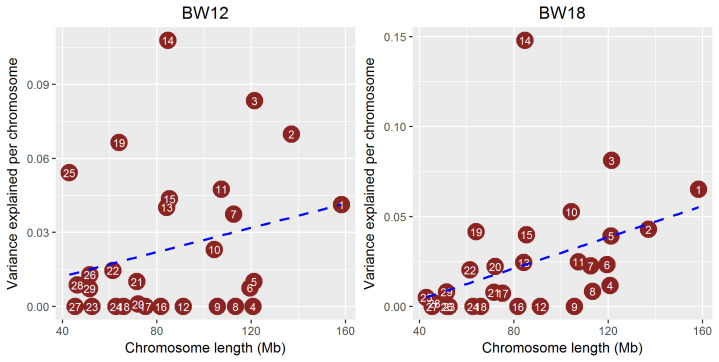
Proportion of genetic variance attributed to each chromosome averaged across body weight traits (12, 18, 24 months) against chromosome length. These proportion values were estimated using a linear mixed model with the restricted maximum likelihood method. The BTA14 accounted for the largest (>10%) portion of the heritability of the three different growth traits, and BTA3 and BTA19 explained moderate (4% to 8%) portion.

We estimated the proportion of genetic variance explained by the common SNPs on each chromosome to determine whether the growth trait was a polygenic characteristic in the Hanwoo population studied. The proportion of total additive genetic variance attributed to each chromosome averaged across three growth traits against chromosome length (Figure 3) was estimated using a linear mixed model with the restricted maximum likelihood method. Figure 3 shows the genetic variance explained by each chromosome. The BTA14 explained >10% of body weight heritability (genetic variance) at 12, 18, and 24 months. BTA3 also had a moderate (≤10%) effect on body weight in Hanwoo cattle. However, the other chromosome had marginal effects on these traits. As for the BTA14 region that identified the largest genetic variance, the genetic variance of the entire BTA14 except for the 25 to 34 Mb region where significant SNPs exist was found to be small (Supplementary Table S2). These results suggest that BTA14 may be a major autosome that affects the entire genome, but since the GWAS is estimated by the LD relationship, there was a limitation in confirming the exact causal variants. We assessed the correlation between heritability explained by each chromosome and chromosome length by calculating the proportion of the genome represented by each chromosome (not including the length of sex chromosomes). The regression line showed a positive correlation between heritability and chromosome length, indicating that each chromosome explained a very small portion of the genetic variance. No gene with a large genetic effect on this trait was observed, but some chromosomes, such as BTA 14, 3, and 19, had moderate genetic effects on the growth traits. Yi et al [16] performed a GWAS to detect QTLs affecting growth traits. That study found 16 significant SNPs for weaning weight (WWT) and 18 significant SNPs for 365-d yearling weight (YWT) in Hanwoo. In that study, a strong signal associated with the weight trait was detected on BTA14, and other traits, such as WWT and YWT, were identified on BTA20. Yang et al [5] revealed that common SNPs explained a large proportion of genetic variance for the height trait in humans. The heritability of human height is around 80%, but that study showed that around 45% of heritability was explained by common SNPs, and the remaining heritability was due to incomplete LD in the human population [5]. Unlike the human population, cattle have a very strong LD structure because of selection. Therefore, BTA14, 3, and 19 have very strong LD structure between marker and causal variants with moderate genetic effects.

### Hierarchical Bayesian mixture model

Given the high density of SNPs, it was impossible to fit all of the SNP markers in the model at the same time. The GWAS used a large number of SNP markers so that the number of parameter estimates was larger than the number of samples. Therefore, we reduced the number of SNP markers using variable selection methods, either by discarding unimportant predictors or by shrinking the marker effect to zero. Among the variable selection methods, Bayesian methods such as the BayesA, B [10], and C [17] models have been developed to very accurately estimate individual SNP effects. Erbe et al [13] developed a new Bayesian method called BayesR, which uses a Bayesian mixed model and the *a priori* assumption of a mixture of a normally distributed mixture of SNP effects.

**Figure 4:**
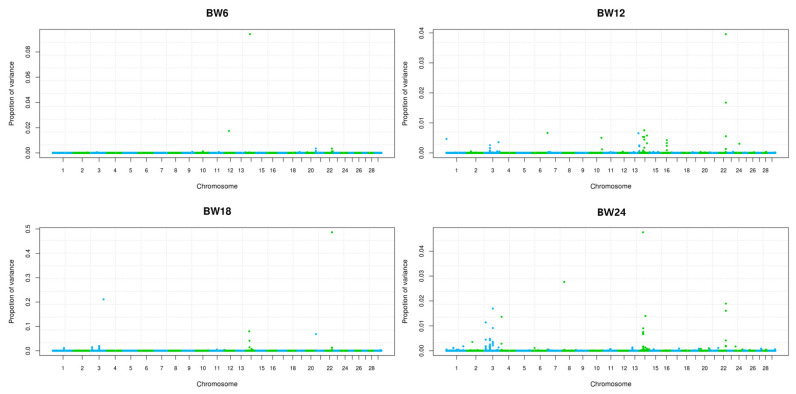
Genome wide proportion of variance for body weight traits at different age (6, 12, 18, and 24 months) using Bayesian mixture model (BayesR) in Hanwoo. The BayesR identified the exact genomic region containing causal SNPs by shrinking marker effect to zero. In these results, six SNP positions on BTA14 were identified same position with the GWAS results, which showed an effect of 0.01% to 0.09%. In BTA22, one SNP position was consistent with the GWAS result, and the effect of this SNP was 0.48%. A high effect SNP of 0.21% was also found in the 96 Mb region of BTA3, but this was not confirmed in the GWAS results. SNP, single nucleotide polymorphism; GWAS, genome-wide association study.

**Figure 5:**
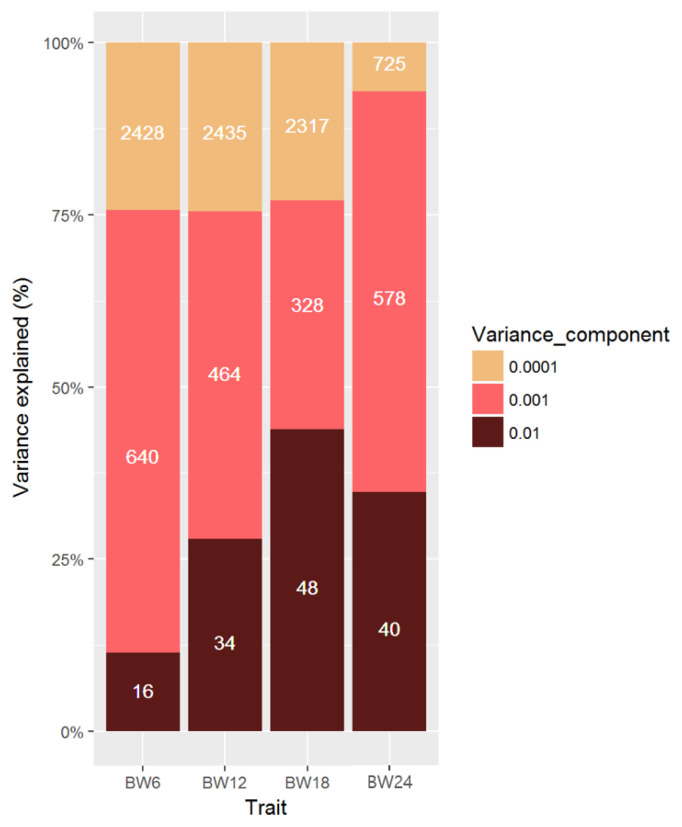
Genetic architecture underlying four traits in Hanwoo using Bayesian mixture model (BayesR). Proportion of additive genetic variation contributed by SNPs with different effect sizes. Many SNPs with small effects (0.0001×***σ***^2^) explained i.e., 25% of BW6, 25% of BW12, 23% of BW18, and 7% of BW24. The moderate effect size (0.001×***σ***^2^) confirmed i.e., 65%, 49%, 30%, and 55% of genetic variation in BW6, 12, 18, and 24, respectively. Therefore, growth trait of Hanwoo can be inferred to controlled by polygenic effect with small and moderate effect size. SNP, single nucleotide polymorphism.

Comparisons between a normal GWAS and BayesR were assessed based on their ability to identify a genomic region of causal variant for growth trait on BTA14 in Hanwoo cattle. Figure 4 shows that BayesR identified the exact genomic region containing causal SNPs on BTA14, 3, and 22. However, the genetic variance explained by each chromosome or SNP was estimated to be a very small proportion against total additive genetic variance (Figure 4). Even SNPs on BTA14 were confirmed six overlapped position with GWAS that had causal effects on growth explained only 0.01% to 0.8% of the genetic variance (Supplementary Table S3). Interestingly, the highest effective SNP for growth trait was confirmed 0.48% of rs42837161 on BTA22 for BW12 to 24, and 0.21% of rs110647998 SNP on BTA3 for BW18 in the research population (Supplementary Table S3). Both areas of BTA3 and 22, which are more effective than BTA14, may need to be monitored for further developmental associations. The proportion of each mixed distribution of SNP markers was estimated for the body weight traits (Figure 5). The proportion of variance explained by each mixed component differed greatly among the four growth traits (Figure 5). In this study, many SNPs with small effects (0.0001×*σ*^2^) explained genetic variance, i.e., 25% of BW6, 25% of BW12, 23% of BW18, and 7% of BW24. The large genetic contribution to the effect size of the SNP produced a moderate effect (0.001×*σ*^2^): 65% of genetic variation in BW6, 49%, 30%, and 55% of genetic variation in BW12, 18, and 24 were explained by SNPs with a moderate effect (0.001×*σ*^2^). These results confirm that the average 70% of genetic variance for small and moderate effects explains the growth traits of Hanwoo cattle. Therefore, growth traits are controlled by many SNPs with small and moderate effect sizes in Hanwoo.

## CONCLUSION

Our results revealed that differences exist in the genetic architecture of different complex traits in Hanwoo cattle. Segregating mutations with a moderate effect on BTA14, 3, and 19 and many other loci with small effects were found for growth traits at different ages. The distribution of the effects was assumed to be normal. The genetic architecture of growth traits will provide important information for predicting the genomic breeding value of animals for selection in the Hanwoo breeding industry.

## Figures and Tables

**Table 1:** Summary statistics for growth traits in Hanwoo

Trait	Mean	SD	Min	Max	Median	h^2^(±SE)
BW6 (kg)	164.3	28.2	69.5	245.0	164.0	0.25±0.09
BW12 (kg)	310.7	33.9	176.0	479.0	311.0	0.32±0.10
BW18 (kg)	475.4	45.3	278.5	655.0	474.5	0.38±0.11
BW24 (kg)	618.0	58.6	361.0	848.5	617.0	0.45±0.11

SD, standard deviation; SE, standard error; BW, body weight of each month age.
