# Supplementary material for: Genomic partitioning of growth traits using a high-density single nucleotide polymorphism array in Hanwoo (Korean cattle)
Source: Asian-Australas J Anim Sci. 2020 Jan 13;33(10):1558–65. doi: 10.5713/ajas.19.0699 (PMC7463090; doi:10.5713/ajas.19.0699)
Supplement: Supplementary file 1 [file ajas-19-0699-suppl.pdf]

**S. Table 1. The significant SNP list by GWAS analysis using GCTA GWAS analysis method**

| Trait | Chromosome | SNP | Physical position | Frequency | SNP effect | Standard error | p-value |
|---|---|---|---|---|---|---|---|
| BW6 | 14 | rs109084660 | 31191960 | 0.228682 | 9.70173 | 1.71836 | 1.64303E-08 |
| BW12 | 14 | rs109080115 | 24656389 | 0.110742 | 16.4653 | 2.98926 | 3.62646E-08 |
| BW12 | 14 | rs109815800 | 25015640 | 0.112957 | 16.7459 | 2.95155 | 1.39828E-08 |
| BW12 | 14 | rs42304759 | 26196375 | 0.125138 | 15.6219 | 2.85461 | 4.43651E-08 |
| BW12 | 14 | rs42765334 | 26328185 | 0.114064 | 16.2173 | 2.97992 | 5.26257E-08 |
| BW12 | 14 | rs109073664 | 26651141 | 0.112957 | 16.397 | 2.983 | 3.86684E-08 |
| BW12 | 14 | rs132748716 | 27050379 | 0.112957 | 16.397 | 2.983 | 3.86684E-08 |
| BW12 | 14 | rs42403984 | 27123573 | 0.112957 | 16.397 | 2.983 | 3.86684E-08 |
| BW12 | 14 | rs133703938 | 27149618 | 0.113511 | 16.3788 | 2.9721 | 3.57076E-08 |
| BW12 | 14 | rs42403970 | 27155254 | 0.112957 | 16.397 | 2.983 | 3.86684E-08 |
| BW12 | 14 | rs109084660 | 31191960 | 0.228682 | 11.3768 | 2.10097 | 6.12699E-08 |
| BW12 | 14 | rs109546980 | 31219729 | 0.10299 | 16.8867 | 3.09038 | 4.64782E-08 |
| BW12 | 14 | rs41619147 | 31377493 | 0.103544 | 16.6997 | 3.08209 | 6.01633E-08 |
| BW12 | 14 | rs109169231 | 34148235 | 0.110742 | 16.7309 | 2.96417 | 1.65772E-08 |
| BW12 | 14 | rs137454864 | 34194915 | 0.110742 | 16.7309 | 2.96417 | 1.65772E-08 |
| BW12 | 14 | rs134630334 | 34264309 | 0.110742 | 16.7309 | 2.96417 | 1.65772E-08 |
| BW12 | 14 | rs110741818 | 35592843 | 0.305094 | 10.2217 | 1.89895 | 7.3327E-08 |
| BW12 | 14 | rs137334521 | 35599284 | 0.295127 | 10.2875 | 1.90164 | 6.3087E-08 |
| BW18 | 14 | rs109080115 | 24656389 | 0.110742 | 23.0032 | 3.95225 | 5.87469E-09 |
| BW18 | 14 | rs109815800 | 25015640 | 0.112957 | 23.5996 | 3.90228 | 1.46966E-09 |
| BW18 | 14 | rs42305768 | 26091728 | 0.383721 | 12.9465 | 2.36936 | 4.65142E-08 |
| BW18 | 14 | rs42304759 | 26196375 | 0.125138 | 21.3415 | 3.77563 | 1.58191E-08 |
| BW18 | 14 | rs42304742 | 26226856 | 0.116279 | 21.81 | 3.87398 | 1.80337E-08 |
| BW18 | 14 | rs42304741 | 26228638 | 0.116833 | 21.1051 | 3.87156 | 5.00014E-08 |
| BW18 | 14 | rs42303720 | 26264142 | 0.116279 | 21.81 | 3.87398 | 1.80337E-08 |
| BW18 | 14 | rs43083557 | 26288440 | 0.115725 | 21.6956 | 3.88835 | 2.41014E-08 |
| BW18 | 14 | rs43083541 | 26302589 | 0.115725 | 21.6956 | 3.88835 | 2.41014E-08 |
| BW18 | 14 | rs42765334 | 26328185 | 0.114064 | 22.2105 | 3.94048 | 1.73548E-08 |
| BW18 | 14 | rs41725166 | 26624399 | 0.119601 | 21.0121 | 3.79901 | 3.18493E-08 |
| BW18 | 14 | rs109073664 | 26651141 | 0.112957 | 21.9882 | 3.94448 | 2.48344E-08 |
| BW18 | 14 | rs132748716 | 27050379 | 0.112957 | 21.9882 | 3.94448 | 2.48344E-08 |
| BW18 | 14 | rs42403984 | 27123573 | 0.112957 | 21.9882 | 3.94448 | 2.48344E-08 |
| BW18 | 14 | rs133703938 | 27149618 | 0.113511 | 21.8575 | 3.93007 | 2.67301E-08 |
| BW18 | 14 | rs42403970 | 27155254 | 0.112957 | 21.9882 | 3.94448 | 2.48344E-08 |
| BW18 | 14 | rs109169231 | 34148235 | 0.110742 | 21.2477 | 3.91999 | 5.94888E-08 |
| BW18 | 14 | rs137454864 | 34194915 | 0.110742 | 21.2477 | 3.91999 | 5.94888E-08 |
| BW18 | 14 | rs134630334 | 34264309 | 0.110742 | 21.2477 | 3.91999 | 5.94888E-08 |
| BW18 | 22 | rs42837161 | 48355980 | 0.156146 | -16.5315 | 3.01268 | 4.0809E-08 |
| BW24 | 14 | rs109080115 | 24656389 | 0.110742 | 33.952 | 5.08971 | 2.54585E-11 |
| BW24 | 14 | rs109815800 | 25015640 | 0.112957 | 33.099 | 5.02503 | 4.49306E-11 |
| BW24 | 14 | rs133319071 | 25544079 | 0.1866 | 22.0657 | 3.93746 | 2.09397E-08 |
| BW24 | 14 | rs42304759 | 26196375 | 0.125138 | 30.5687 | 4.8664 | 3.35155E-10 |
| BW24 | 14 | rs42304742 | 26226856 | 0.116279 | 31.7272 | 4.98999 | 2.04182E-10 |
| BW24 | 14 | rs42304741 | 26228638 | 0.116833 | 30.7343 | 4.98677 | 7.13018E-10 |
| BW24 | 14 | rs42303720 | 26264142 | 0.116279 | 31.7272 | 4.98999 | 2.04182E-10 |
| BW24 | 14 | rs43083557 | 26288440 | 0.115725 | 31.7633 | 5.00868 | 2.27326E-10 |
| BW24 | 14 | rs43083541 | 26302589 | 0.115725 | 31.7633 | 5.00868 | 2.27326E-10 |
| BW24 | 14 | rs42765334 | 26328185 | 0.114064 | 32.3123 | 5.07619 | 1.94691E-10 |
| BW24 | 14 | rs134721150 | 26603341 | 0.122924 | 27.4034 | 4.76684 | 8.99011E-09 |
| BW24 | 14 | rs41725183 | 26604168 | 0.122924 | 27.4034 | 4.76684 | 8.99011E-09 |
| BW24 | 14 | rs41725166 | 26624399 | 0.119601 | 30.5618 | 4.89234 | 4.18746E-10 |
| BW24 | 14 | rs109073664 | 26651141 | 0.112957 | 32.0692 | 5.08111 | 2.76405E-10 |
| BW24 | 14 | rs111021769 | 26662633 | 0.138427 | 24.4208 | 4.52004 | 6.56098E-08 |
| BW24 | 14 | rs41724536 | 26766010 | 0.11794 | 28.5482 | 4.94706 | 7.89187E-09 |
| BW24 | 14 | rs41724015 | 26794576 | 0.120709 | 27.1519 | 4.93392 | 3.73166E-08 |
| BW24 | 14 | rs42404027 | 26997876 | 0.138981 | 28.2527 | 4.68724 | 1.66434E-09 |
| BW24 | 14 | rs42404006 | 27035971 | 0.138981 | 28.2527 | 4.68724 | 1.66434E-09 |
| BW24 | 14 | rs132748716 | 27050379 | 0.112957 | 32.0692 | 5.08111 | 2.76405E-10 |
| BW24 | 14 | rs42403984 | 27123573 | 0.112957 | 32.0692 | 5.08111 | 2.76405E-10 |
| BW24 | 14 | rs133703938 | 27149618 | 0.113511 | 31.8133 | 5.06258 | 3.29975E-10 |
| BW24 | 14 | rs42403970 | 27155254 | 0.112957 | 32.0692 | 5.08111 | 2.76405E-10 |
| BW24 | 14 | rs42893390 | 27271835 | 0.107973 | 29.7253 | 5.15237 | 7.96227E-09 |
| BW24 | 14 | rs109084660 | 31191960 | 0.228682 | 20.9096 | 3.57123 | 4.76985E-09 |
| BW24 | 14 | rs109546980 | 31219729 | 0.10299 | 30.3616 | 5.26119 | 7.88646E-09 |
| BW24 | 14 | rs41619147 | 31377493 | 0.103544 | 30.1172 | 5.24681 | 9.46215E-09 |
| BW24 | 14 | rs109169231 | 34148235 | 0.110742 | 30.8979 | 5.05098 | 9.52255E-10 |
| BW24 | 14 | rs137454864 | 34194915 | 0.110742 | 30.8979 | 5.05098 | 9.52255E-10 |
| BW24 | 14 | rs134630334 | 34264309 | 0.110742 | 30.8979 | 5.05098 | 9.52255E-10 |
| BW24 | 6 | rs43457593 | 39932557 | 0.03433 | 48.6504 | 8.90798 | 4.72281E-08 |
| BW24 | 6 | rs133886965 | 42404901 | 0.0310078 | 52.8215 | 9.72707 | 5.62388E-08 |
| BW24 | 14 | rs109517898 | 46359424 | 0.371539 | 17.6028 | 3.13551 | 1.97709E-08 |
| BW24 | 14 | rs110705278 | 46392030 | 0.374308 | 17.603 | 3.13195 | 1.90467E-08 |

S.Table 2. The window size-based chromosoe partitioning analysis in a BTA14 chromosome

| Trait | BTA14 whole | | Without_specific_window | |
|---|---|---|---|---|
| | Variance | Standard error | Variance | Standard error |
| BW6_heritability | 0.252956 | 0.050387 | 0.207181 | 0.04827 |
| BW12_heritability | 0.226079 | 0.046968 | 0.199983 | 0.045116 |
| BW18_heritability | 0.25732 | 0.048191 | 0.225776 | 0.046128 |
| BW23_heritability | 0.282821 | 0.04945 | 0.248769 | 0.047686 |
| BW6_V(G) | 202.406644 | 46.202151 | 164.779387 | 42.54753 |
| BW12_V(G) | 265.824201 | 62.06686 | 235.638772 | 58.717011 |
| BW18_V(G) | 530.564003 | 114.520453 | 465.54937 | 107.198109 |
| BW23_V(G) | 989.44875 | 203.674133 | 872.117723 | 191.872437 |

**S. Table 3. The proportion of variance for three type of growth traits using Bayesian mixture model (BayesR)**

| Trait | Chromosome | Reference ID | Physical position | Effect size |
|---|---|---|---|---|
| BW6 | 12 | rs109360887 | 31368562 | 0.017277151 |
| BW6 | 14 | rs109084660 | 31191960 | 0.094032164 |
| BW12 | 22 | rs42837161 | 48355980 | 0.039585091 |
| BW12 | 22 | rs42466723 | 48376462 | 0.016792892 |
| BW18 | 1 | rs136100553 | 88680006 | 0.011192991 |
| BW18 | 3 | rs109305070 | 7188354 | 0.014680347 |
| BW18 | 3 | rs41624536 | 60324610 | 0.019426537 |
| BW18 | 3 | rs110806975 | 60325941 | 0.014484642 |
| BW18 | 3 | rs110647998 | 96628948 | 0.211257814 |
| BW18 | 14 | rs109815800 | 25015640 | 0.079857982 |
| BW18 | 14 | rs42403984 | 27123573 | 0.014226019 |
| BW18 | 14 | rs133703938 | 27149618 | 0.040847078 |
| BW18 | 21 | rs132786958 | 2625626 | 0.068327455 |
| BW18 | 22 | rs42837161 | 48355980 | 0.486633282 |
| BW18 | 22 | rs42466723 | 48376462 | 0.013014708 |
| BW24 | 3 | rs109754311 | 7184922 | 0.01136204 |
| BW24 | 3 | rs135142468 | 60322269 | 0.016959451 |
| BW24 | 4 | rs134745551 | 5539434 | 0.013608531 |
| BW24 | 8 | rs137525627 | 11105358 | 0.027639413 |
| BW24 | 14 | rs109080115 | 24656389 | 0.047587789 |
| BW24 | 14 | rs110705278 | 46392030 | 0.013945498 |
| BW24 | 22 | rs42837161 | 48355980 | 0.016071794 |
| BW24 | 22 | rs42837153 | 48364982 | 0.018902995 |
